# Supplementary material for: Impact of the COVID-19 Pandemic on Patients with Parkinson’s Disease from the Perspective of Treating Physicians—A Nationwide Cross-Sectional Study
Source: Brain Sci. 2022 Mar 5;12(3):353. doi: 10.3390/brainsci12030353 (PMC8946104; doi:10.3390/brainsci12030353)
Supplement: Supplementary file 1 [file brainsci-12-00353-s001.zip › Supplementary Material.pdf]

Supplementary Material

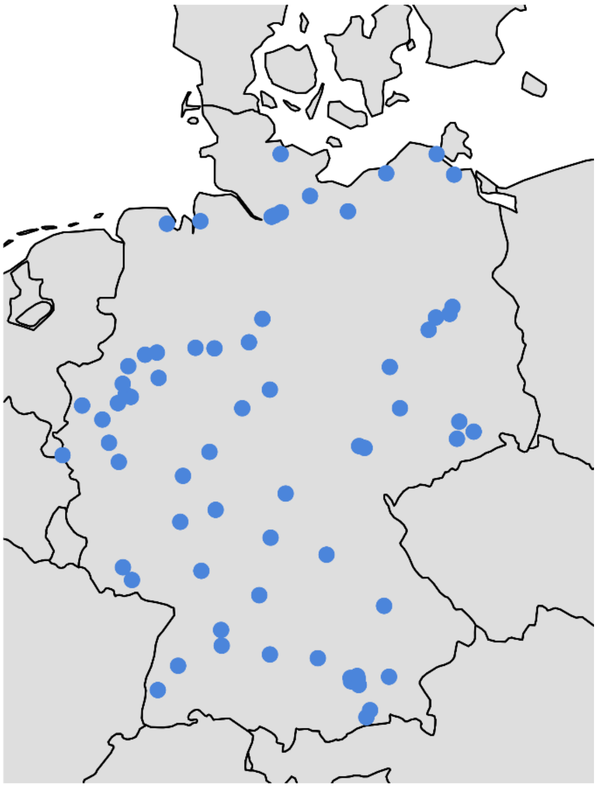

Supplementary Figure S1: Map of Germany displaying the location of all 67 healthcare facilities contacted for this study.

Supplementary Table S1: Complete survey results.

1. Please indicate which specialization you have.		
Neurologist	39	97.5%
Internist	0	0.0%
Physician in other specialty	1	2.5%
2. I am a family physician/general practitioner.		
Yes	0	0.0%
No	40	100.0%
3. In which sector of patient care do you work?		
Private office	3	7.5%
Rehabilitation clinic	0	0.0%
Hospital	30	75.0%
Other: if so, please fill in	7	17.5%
Hospital and private office	2	5.0%
Hospital and outpatient department	1	2.5%
Outpatient department	2	5.0%
„Parkinson-center“	1	2.5%
DBS outpatient department	1	2.5%
4. Which statement best applies to you?		
I do NOT treat Parkinson's patients.	0	0.0%
I RARELY treat patients with Parkinson's disease.	2	5.0%
I REGULARY treat patients with Parkinson's disease.	8	20.0%

Parkinson's patients are my MAIN FOCUS, but I treat other patients as well.	29	72.5%
I EXCLUSIVELY treat Parkinson's patients.	1	2.5%
5. If you compare what you do now to before the pandemic: How often did you treat Parkinson's patients?		
Less often	17	42.5%
Equally often	20	50.0%
More often	3	7.5%
6. If you compare your activity now with the time before the pandemic: Did you have to cancel any of your patients' appointments due to the pandemic?		
No	5	12.5%
Yes (few)	20	50.0%
Yes (many)	15	37.5%
Yes (all)	0	0.0%
7. If you compare your work now with the time before the pandemic: Did patients cancel appointments of their own accord due to the pandemic?		
No	1	2.5%
Yes (few)	25	62.5%
Yes (many)	14	35.0%
Yes (all)	0	0.0%
8. If you compare your work now with the time before the pandemic: Do you feel your patients received worse care during the pandemic?		
No	1	2.5%
Yes (few)	20	50.0%
Yes (many)	19	47.5%
Yes (all)	0	0.0%
9. If you compare your work now with the time before the pandemic: Do you have the feeling that the symptoms of your patients are getting worse because of the pandemic?		
No	7	17.5%
Yes (for a few)	24	60.0%
Yes (for many)	9	22.5%
Yes (for all)	0	0.0%
10. If you think of all your Parkinson's patients: Do you feel that the pandemic and associated changes resulted in a change in certain symptoms?<br><br>10.1 Motor symptoms (rigor, tremor, bradykinesia, OFF-time, dyskinesia).		
Worsened for many	5	12.5%
Worsened for some	25	62.5%
Unchanged	10	25.0%
Improved for some	0	0.0%
Improved for many	0	0.0%
10.2 Neuropsychiatric symptoms (depression, anxiety, obsessive compulsive behaviour, hallucinations)		
Worsened for many	11	27.5%
Worsened for some	22	55.0%

Unchanged	7	17.5%
Improved for some	0	0.0%
Improved for many	0	0.0%
10.3 Autonomic symptoms (constipation, urinary incontinence, orthostatic dysregulation)		
Worsened for many	0	0.0%
Worsened for some	9	23.7%
Unchanged	29	76.3%
Improved for some	0	0.0%
Improved for many	0	0.0%
10.4 Sleep disturbances		
Worsened for many	2	5.3%
Worsened for some	25	65.8%
Unchanged	11	28.9%
Improved for some	0	0.0%
Improved for many	0	0.0%
11. Did you have problems organizing the following therapies for your patients during the pandemic?		
11.1 Rehabilitation measures		
No difficulties	2	5.4%
Rare difficulties	14	37.8%
Frequent difficulties	21	56.8%
Impossible	0	0.0%
11.2 Physiotherapy or occupational therapy		
No difficulties	3	7.5%
Rare difficulties	14	35.0%
Frequent difficulties	23	57.5%
Impossible	0	0.0%
11.3 Speech therapy		
No difficulties	2	5.0%
Rare difficulties	13	32.5%
Frequent difficulties	25	62.5%
Impossible	0	0.0%
11.4 Endoscopic intervention (e.g.: implantation of a jejunal probe)		
No difficulties	12	32.4%
Rare difficulties	17	45.9%
Frequent difficulties	8	21.6%
Impossible	0	0.0%
11.5 Deep brain stimulation (DBS) surgery		
No difficulties	11	28.9%
Rare difficulties	11	28.9%
Frequent difficulties	15	39.5%
Impossible	1	2.6%
11.6 Referrals to a hospital		
No difficulties	6	16.2%
Rare difficulties	22	59.5%
Frequent difficulties	9	24.3%
Impossible	0	0.0%

12. Did you have to close your practice/department (partially) due to the pandemic?		
No	10	25.0%
Yes (partially)	28	70.0%
Yes (completely)	2	5.0%
13. Did you have to accept financial losses due to the pandemic?		
None	18	48.6%
Minor	15	40.5%
Major	4	10.8%
14. Did you have additional expenditures due to the pandemic?		
None	25	65.8%
Minor	12	31.6%
Major	1	2.6%
15. Do hygiene concepts in your department/practice mean extra work for you?		
None	1	2.5%
Minor	16	40.0%
Major	23	57.5%
16.1 Have you expanded your services due to the pandemic?		
Through increased home consultations		
Yes	5	12.8%
No	34	87.2%
17.1 How were these services accepted by your PD patients?		
Not accepted	0	0.0%
Somewhat accepted	3	60.0%
Broadly accepted	2	40.0%
16.2 Have you expanded your services due to the pandemic?		
Through online offers / consultation hours		
Yes	25	64.1%
No	14	35.9%
17.2 How were these services accepted by your PD patients?		
Not accepted	1	4.0%
Somewhat accepted	20	80.0%
Broadly accepted	4	16.0%
16.3 Have you expanded your services due to the pandemic?		
Through telephone consultations		
Yes	31	77.5%
No	9	22.5%
17.3 How were these services accepted by your PD patients?		
Not accepted	0	0.0%
Somewhat accepted	14	45.2%
Broadly accepted	17	54.8%
16.4 Have you expanded your services due to the pandemic?		
By providing written information to your patients		
Yes	6	15.4%
No	33	84.6%
17.4 How were these services accepted by your PD patients?		
Not accepted	0	0.0%
Somewhat accepted	5	83.3%
Broadly accepted	1	16.7%
16.5 Have you expanded your services due to the pandemic?		

Through other measures		
Yes (Please name the measures: _____________)	3	7.5%
Homepage, social media	1	2.5%
Emergency consultation hour	1	2.5%
No	37	92.5%
17.5 How were these services accepted by your PD patients?		
Not accepted	0	0.0%
Somewhat accepted	0	0.0%
Broadly accepted	2	100.0%
18. Have you treated/visited patients with COVID19 yourself?		
No	15	37.5%
Yes	25	62.5%
19. Do you feel that your health is at risk during your work?		
No	18	45.0%
Yes	22	55.0%
20. Do you feel protected by the protective measures available to you?		
No	0	0.0%
Yes (somewhat protected)	17	42.5%
Yes (very well protected)	23	57.5%
21. Did you find your work with PD patients more stressful/demanding during the pandemic compared to before the pandemic?		
No	24	60.0%
Yes	16	40.0%
22. Did your quality of care subjectively deteriorate?		
No	25	62.5%
Yes (slightly)	15	37.5%
Yes (significantly)	0	0.0%
23. Have you been vaccinated against influenza?		
Yes	33	82.5%
No	7	17.5%
24. If a vaccine against SARSCoV2 were available, would you get vaccinated?		
Yes	39	97.5%
No	1	2.5%
25. Why would you not get vaccinated?		
I don't think vaccinating is generally a good idea.	0	0.0%
I do not think I could contract COVID-19.	0	0.0%
I do not trust a new vaccine yet.	1	100.0%
I am afraid of side effects.	0	0.0%
Other reasons: _________	0	0.0%
26. Would you recommend that your Parkinson's patients get vaccinated?		
Yes	40	100.0%
No	0	0.0%

Supplementary Table S2: Transcripts of the German and English version of the ParCoPa survey, physicians' part.

German transcript of the ParCoPa survey (physicians)	English transcript of the ParCoPa survey (physicians)
1. Bitte geben Sie an, welche Spezialisierung Sie haben.	1. Please indicate which specialization you have.
Neurologin/Neurologe oder Nervenarzt/ärztin	Neurologist
Internist/Internistin	Internist
Facharzt/ärztin für andere Fachrichtung	Physician in other specialty
2. Ich bin Hausarzt/Hausärztin.	2. I am a family physician/general practitioner.
Ja	Yes
Nein	No
3. In welchem Sektor der Patientenversorgung arbeiten Sie?	3. In which sector of patient care do you work?
Praxis	Private office
Rehabilitationsklinik	Rehabilitation clinic
Akutkrankenhaus	Hospital
Sonstiges: dann bitte ausfüllen	Other: if so, please fill in
4. Was trifft auf Sie am besten zu?	4. Which statement best applies to you?
Ich behandele KEINE Parkinsonpatienten.	I do NOT treat Parkinson's patients.
Ich behandele SELTEN Parkinsonpatienten.	I RARELY treat patients with Parkinson's disease.
Ich behandele REGELMAESSIG Parkinsonpatienten.	I REGULARY treat patients with Parkinson's disease.
Parkinsonpatienten sind mein SCHWERPUNKT, aber ich behandele auch andere Patienten.	Parkinson's patients are my MAIN FOCUS, but I treat other patients as well.
Ich behandele AUSSCHLIESSLICH Parkinsonpatienten	I EXCLUSIVELY treat Parkinson's patients.
5. Wenn Sie Ihre Tätigkeit jetzt mit der Zeit VOR der Pandemie vergleichen: Wie oft haben Sie Parkinsonpatienten behandelt?	5. If you compare what you do now to before the pandemic: How often did you treat Parkinson's patients?
Seltener	Less often
Gleich oft	Equally often
Häufiger	More often
6. Wenn Sie Ihre Tätigkeit jetzt mit der Zeit VOR der Pandemie vergleichen: Mussten Sie Termine Ihrer Patienten/innen auf Grund der Pandemie absagen?	6. If you compare your activity now with the time before the pandemic: Did you have to cancel any of your patients' appointments due to the pandemic?
Nein	No
Ja (wenige)	Yes (few)
Ja (viele)	Yes (many)
Ja (alle)	Yes (all)
7. Wenn Sie Ihre Tätigkeit jetzt mit der Zeit VOR der Pandemie vergleichen: Haben Patienten/innen von sich aus auf Grund der Pandemie Termine abgesagt?	7. If you compare your work now with the time before the pandemic: Did patients cancel appointments of their own accord due to the pandemic?

Deutsch	English
Nein	No
Ja (wenige)	Yes (few)
Ja (viele)	Yes (many)
Ja (alle)	Yes (all)
8. Wenn Sie Ihre Tätigkeit jetzt mit der Zeit VOR der Pandemie vergleichen: Haben Sie das Gefühl Ihre Patienten/innen sind während der Pandemie schlechter versorgt?	8. If you compare your work now with the time before the pandemic: Do you feel your patients received worse care during the pandemic?
Nein	No
Ja (wenige)	Yes (few)
Ja (viele)	Yes (many)
Ja (alle)	Yes (all)
9. Wenn Sie Ihre Tätigkeit jetzt mit der Zeit VOR der Pandemie vergleichen: Haben Sie das Gefühl die Symptomatik Ihrer Patienten/innen verschlechtere sich wegen der Pandemie?	9. If you compare your work now with the time before the pandemic: Do you have the feeling that the symptoms of your patients are getting worse because of the pandemic?
Nein	No
Ja (bei wenigen)	Yes (for a few)
Ja (bei vielen)	Yes (for many)
Ja (bei allen)	Yes (for all)
10. Wenn Sie sich ein Bild über alle Ihre Parkinsonpatienten machen: Haben Sie den Eindruck, dass die Pandemie und die damit verbundenen Veränderungen zu einer Veränderung bestimmter Symptome führte?	10. If you think of all your Parkinson's patients: Do you feel that the pandemic and associated changes resulted in a change in certain symptoms?
10.1 Motorische Symptome (Rigor, Tremor, Bradykinese, OFF-Zeit, Dyskinesien)	10.1 Motor symptoms (rigor, tremor, bradykinesia, OFF-time, dyskinesia).
Bei vielen verschlechtert	Worsened for many
Bei einigen verschlechtert	Worsened for some
Nicht verändert	Unchanged
Bei einigen gebessert	Improved for some
Bei vielen gebessert	Improved for many
10.2 Neuropsychiatrische Symptome (Depressivität, Angst, Zwangsverhalten, Halluzinationen)	10.2 Neuropsychiatric symptoms (depression, anxiety, obsessive compulsive behaviour, hallucinations)
Bei vielen verschlechtert	Worsened for many
Bei einigen verschlechtert	Worsened for some
Nicht verändert	Unchanged
Bei einigen gebessert	Improved for some
Bei vielen gebessert	Improved for many
10.3 Autonome Symptome (Obstipation, Urininkontinenz, orthostatische Dysregulation)	10.3 Autonomic symptoms (constipation, urinary incontinence, orthostatic dysregulation)
Bei vielen verschlechtert	Worsened for many
Bei einigen verschlechtert	Worsened for some
Nicht verändert	Unchanged
Bei einigen gebessert	Improved for some
Bei vielen gebessert	Improved for many
10.4 Schlafstörungen	10.4 Sleep disturbances
Bei vielen verschlechtert	Worsened for many
Bei einigen verschlechtert	Worsened for some

Deutsch	English
Nicht verändert	Unchanged
Bei einigen gebessert	Improved for some
Bei vielen gebessert	Improved for many
11. Hatten Sie Probleme, während der Pandemie folgende Therapien für Ihre Patienten/innen zu organisieren?	11. Did you have problems organizing the following therapies for your patients during the pandemic?
11.1 Rehabilitative Maßnahmen	11.1 Rehabilitation measures
Keine Probleme	No difficulties
Selten Probleme	Rare difficulties
Oft Probleme	Frequent difficulties
Unmöglich	Impossible
11.2 Physio/Ergotherapie	11.2 Physiotherapy or occupational therapy
Keine Probleme	No difficulties
Selten Probleme	Rare difficulties
Oft Probleme	Frequent difficulties
Unmöglich	Impossible
11.3 Logopädie	11.3 Speech therapy
Keine Probleme	No difficulties
Selten Probleme	Rare difficulties
Oft Probleme	Frequent difficulties
Unmöglich	Impossible
11.4 Endoskopische Untersuchungen (z.B.: Implantation einer Jejunalsonde)	11.4 Endoscopic intervention (e.g.: implantation of a jejunal probe)
Keine Probleme	No difficulties
Selten Probleme	Rare difficulties
Oft Probleme	Frequent difficulties
Unmöglich	Impossible
11.5 DBS-Operation (Tiefe Hirnstimulation)	11.5 DBS surgery (Deep Brain Stimulation)
Keine Probleme	No difficulties
Selten Probleme	Rare difficulties
Oft Probleme	Frequent difficulties
Unmöglich	Impossible
11.6 Krankenhauseinweisung	11.6 Referrals to a hospital
Keine Probleme	No difficulties
Selten Probleme	Rare difficulties
Oft Probleme	Frequent difficulties
Unmöglich	Impossible
12. Mussten Sie Ihre Praxis/Abteilung (teilweise) durch die Pandemie schließen?	12. Did you have to close your practice/department (partially) due to the pandemic?
Nein	No
Ja (teilweise)	Yes (partially)
Ja (komplett)	Yes (completely)
13. Mussten Sie auf Grund der Pandemie finanzielle Einbußen hinnehmen?	13. Did you have to accept financial losses due to the pandemic?
Keine	None
Geringere	Minor
Deutliche	Major
14. Mussten Sie auf Grund der Pandemie Investitionen tätigen?	14. Did you have additional expenditures due to the pandemic?

German	English
Keine	None
Geringere	Minor
Deutliche	Major
15. Bedeuten Hygienekonzepte in Ihrer Abteilung/Praxis Mehrarbeit für Sie?	15. Do hygiene concepts in your department/practice mean extra work for you?
Keine	None
Geringere	Minor
Deutliche	Major
16.1 Haben Sie Ihr Angebot aufgrund der Pandemie erweitert?	16.1 Have you expanded your services due to the pandemic?
Durch vermehrte häusliche Konsultationen	Through increased home consultations
Ja	Yes
Nein	No
17.1 Wie wurden diese Angebote von Ihren Parkinsonpatienten genutzt?	17.1 How were these services accepted by your PD patients?
Nicht genutzt	Not accepted
Wenig genutzt	Somewhat accepted
Viel genutzt	Broadly accepted
16.2 Haben Sie Ihr Angebot aufgrund der Pandemie erweitert?	16.2 Have you expanded your services due to the pandemic?
Durch Onlineangebote/Sprechstunden	Through online offers / consultation hours
Ja	Yes
Nein	No
17.2 Wie wurden diese Angebote von Ihren Parkinsonpatienten genutzt?	17.2 How were these services accepted by your PD patients?
Nicht genutzt	Not accepted
Wenig genutzt	Somewhat accepted
Viel genutzt	Broadly accepted
16.3 Haben Sie Ihr Angebot aufgrund der Pandemie erweitert?	16.3 Have you expanded your services due to the pandemic?
Durch telefonische Sprechstunden	Through telephone consultations
Ja	Yes
Nein	No
17.3 Wie wurden diese Angebote von Ihren Parkinsonpatienten genutzt?	17.3 How were these services accepted by your PD patients?
Nicht genutzt	Not accepted
Wenig genutzt	Somewhat accepted
Viel genutzt	Broadly accepted
16.4 Haben Sie Ihr Angebot aufgrund der Pandemie erweitert?	16.4 Have you expanded your services due to the pandemic?
Durch schriftliche Informationen für Ihre Patienten/innen	By providing written information to your patients
Ja	Yes
Nein	No
17.4 Wie wurden diese Angebote von Ihren Parkinsonpatienten genutzt?	17.4 How were these services accepted by your PD patients?
Nicht genutzt	Not accepted
Wenig genutzt	Somewhat accepted
Viel genutzt	Broadly accepted
16.5 Haben Sie Ihr Angebot aufgrund der Pandemie erweitert?	16.5 Have you expanded your services due to the pandemic?

Durch andere Maßnahmen	Through other measures
Ja (Bitte benennen Sie die Maßnahmen: ______________)	Yes (Please name the measures: ____________)
Nein	No
17.5 Wie wurden diese Angebote von Ihren Parkinsonpatienten genutzt?	17.5 How were these services accepted by your PD patients?
Nicht genutzt	Not accepted
Wenig genutzt	Somewhat accepted
Viel genutzt	Broadly accepted
18. Haben Sie selbst an COVID19 Erkrankte behandelt/visitiert?	18. Have you treated/visited patients with COVID19 yourself?
Nein	No
Ja	Yes
19. Fühlen Sie sich gesundheitlich gefährdet während Ihrer Arbeit?	19. Do you feel that your health is at risk during your work?
Nein	No
Ja	Yes
20. Fühlen Sie sich durch die Ihnen zur Verfügung stehenden Schutzmaßnahmen geschützt?	20. Do you feel protected by the protective measures available to you?
Nein	No
Ja (ein wenig geschützt)	Yes (somewhat protected)
Ja (sehr gut geschützt)	Yes (very well protected)
21. Haben Sie Ihre Arbeit mit Parkinsonpatienten während der Pandemie als stressiger/fordernder im Vergleich zu der Zeit vor der Pandemie empfunden?	21. Did you find your work with PD patients more stressful/demanding during the pandemic compared to before the pandemic?
Nein	No
Ja	Yes
22. Hat sich Ihre Behandlungsqualität subjektiv verschlechtert?	22. Did your quality of care subjectively deteriorate?
Nein	No
Ja (ein wenig)	Yes (slightly)
Ja (deutlich)	Yes (significantly)
23. Sind Sie gegen Influenza geimpft?	23. Have you been vaccinated against influenza?
Ja	Yes
Nein	No
24. Würden Sie sich, sollte ein Impfstoff gegen SARSCoV2 verfügbar sein, impfen lassen?	24. If a vaccine against SARSCoV2 were available, would you get vaccinated?
Ja	Yes
Nein	No
25. Warum würden Sie sich nicht impfen lassen?	25. Why would you not get vaccinated?
Ich finde impfen generell nicht sinnvoll	I don't think vaccinating is generally a good idea.
Ich denke nicht, dass ich mich an COVID-19 anstecken könnte.	I do not think I could contract COVID-19.
Ich vertraue einem neuen Impfstoff noch nicht.	I do not trust a new vaccine yet.

Ich habe Angst vor Nebenwirkungen.	I am afraid of side effects.
Andere Gründe: _________	Other reasons: ________
26. Würden Sie Ihren Parkinsonpatienten empfehlen, sich impfen zu lassen?	26. Would you recommend that your Parkinson's patients get vaccinated?
Ja	Yes
Nein	No

Supplementary Table S3

Checklist for Reporting Of Survey Studies (CROSS)

Section/topic	Item	Item description	Reported on page #
**Title and abstract**			
Title and abstract	1a	State the word "survey" along with a commonly used term in title or abstract to introduce the study's design.	Abstract
	1b	Provide an informative summary in the abstract, covering background, objectives, methods, findings/results, interpretation/discussion, and conclusions.	Abstract
**Introduction**			
Background	2	Provide a background about the rationale of study, what has been previously done, and why this survey is needed.	Page 1
Purpose/aim	3	Identify specific purposes, aims, goals, or objectives of the study.	Page 2
**Methods**			
Study design	4	Specify the study design in the methods section with a commonly used term (e.g., cross-sectional or longitudinal).	Page 2
Data collection methods	5a	Describe the questionnaire (e.g., number of sections, number of questions, number and names of instruments used).	Page 2
	5b	Describe all questionnaire instruments that were used in the survey to measure particular concepts. Report target population, reported validity and reliability information, scoring/classification procedure, and reference links (if any).	Page 2
	5c	Provide information on pretesting of the questionnaire, if performed (in the article or in an online supplement). Report the method of pretesting, number of times questionnaire was pre-tested, number and demographics of participants used for pretesting, and the level of similarity of demographics between pre-testing participants and sample population.	Page 2
	5d	Questionnaire if possible, should be fully provided (in the article, or as appendices or as an online supplement).	Supplements
Sample characteristics	6a	Describe the study population (i.e., background, locations, eligibility criteria for participant inclusion in survey, exclusion criteria).	Page 2

	6b	Describe the sampling techniques used (e.g., single stage or multistage sampling, simple random sampling, stratified sampling, cluster sampling, convenience sampling). Specify the locations of sample participants whenever clustered sampling was applied.	NA
	6c	Provide information on sample size, along with details of sample size calculation.	Page 3
	6d	Describe how representative the sample is of the study population (or target population if possible), particularly for population-based surveys.	Page 3
	7a	Provide information on modes of questionnaire administration, including the type and number of contacts, the location where the survey was conducted (e.g., outpatient room or by use of online tools, such as SurveyMonkey).	Page 2
Survey administration	7b	Provide information of survey's time frame, such as periods of recruitment, exposure, and follow-up days.	Page 2
	7c	Provide information on the entry process:<br>–>For non-web-based surveys, provide approaches to minimize human error in data entry.<br>–>For web-based surveys, provide approaches to prevent "multiple participation" of participants.	Page 2
Study preparation	8	Describe any preparation process before conducting the survey (e.g., interviewers' training process, advertising the survey).	Page 2
Ethical considerations	9a	Provide information on ethical approval for the survey if obtained, including informed consent, institutional review board [IRB] approval, Helsinki declaration, and good clinical practice [GCP] declaration (as appropriate).	Page 2
	9b	Provide information about survey anonymity and confidentiality and describe what mechanisms were used to protect unauthorized access.	Page 2
	10a	Describe statistical methods and analytical approach. Report the statistical software that was used for data analysis.	Page 2
	10b	Report any modification of variables used in the analysis, along with reference (if available).	Page 2
	10c	Report details about how missing data was handled. Include rate of missing items, missing data mechanism (i.e., missing completely at random [MCAR], missing at random [MAR] or missing not at random [MNAR]) and methods used to deal with missing data (e.g., multiple imputation).	Page 2
Statistical analysis	10d	State how non-response error was addressed.	NA
	10e	For longitudinal surveys, state how loss to follow-up was addressed.	NA
	10f	Indicate whether any methods such as weighting of items or propensity scores have been used to adjust for non-representativeness of the sample.	NA
	10g	Describe any sensitivity analysis conducted.	NA

**Results**			
Respondent characteristics	11a	Report numbers of individuals at each stage of the study. Consider using a flow diagram, if possible.	Page 3
	11b	Provide reasons for non-participation at each stage, if possible.	NA
	11c	Report response rate, present the definition of response rate or the formula used to calculate response rate.	Page 1
	11d	Provide information to define how unique visitors are determined. Report number of unique visitors along with relevant proportions (e.g., view proportion, participation proportion, completion proportion).	NA
Descriptive results	12	Provide characteristics of study participants, as well as information on potential confounders and assessed outcomes.	Page 2
Main findings	13a	Give unadjusted estimates and, if applicable, confounder-adjusted estimates along with 95% confidence intervals and p-values.	Page 3
	13b	For multivariable analysis, provide information on the model building process, model fit statistics, and model assumptions (as appropriate).	Page 2
	13c	Provide details about any sensitivity analysis performed. If there are considerable amount of missing data, report sensitivity analyses comparing the results of complete cases with that of the imputed dataset (if possible).	NA
**Discussion**			
Limitations	14	Discuss the limitations of the study, considering sources of potential biases and imprecisions, such as non-representativeness of sample, study design, important uncontrolled confounders.	Page 6
Interpretations	15	Give a cautious overall interpretation of results, based on potential biases and imprecisions and suggest areas for future research.	Page 6
Generalizability	16	Discuss the external validity of the results.	Page 6
**Other sections**			
Role of funding source	17	State whether any funding organization has had any roles in the survey's design, implementation, and analysis.	Page 7
Conflict of interest	18	Declare any potential conflict of interest.	Page 7
Acknowledgements	19	Provide names of organizations/persons that are acknowledged along with their contribution to the research.	Page 7
