# Supplementary material for: Impact of the COVID-19 Pandemic on Patients with Parkinson’s Disease from the Perspective of Treating Physicians—A Nationwide Cross-Sectional Study
Source: Brain Sci. 2022 Mar 5;12(3):353. doi: 10.3390/brainsci12030353 (PMC8946104; doi:10.3390/brainsci12030353)
Supplement: Supplementary file 1 [file brainsci-12-00353-s001.zip › Wolff et al. 2022 Questionnaire Annotations.pdf]

ParCoPa Behandelndeninformation - Bestätigung Einwilligung mnpparcpprelimb

{ IASHorizontalGroup: .... Standard - horizontal }

Sind Sie volljährig?

Sie dürfen nur an dieser Umfrage teilnehmen, wenn Sie volljährig (mindestens 18 Jahre) alt sind.

Kommentar

Sind Sie volljährig?

◯ Ja (1 - 210099) ◯ Nein (0 - 210118)

prelimbv {Horizontal-Radiobutton}

{ IASHeadline: .... Überschrift }

Einverständniserklärung

{ IASHorizontalGroup: .... Standard - horizontal }

Ich erkläre mein Einverständnis

Ich erkläre mein Einverständnis:

Kommentar

Ich bestätige, dass ich mich über das geplante Vorgehen im Rahmen der Studie ParCoPa ausführlich aufgeklärt fühle. Ich hatte genügend Zeit mich zu entscheiden.

Ich verstehe, dass meine Teilnahme freiwillig ist und dass ich jederzeit ohne Angabe von Gründen aus der Studie austreten kann. Durch eine Beendigung entstehen mir keinerlei Nachteile bezüglich meiner medizinischen Versorgung.

Mir ist bekannt, dass im Rahmen dieses Forschungsvorhabens personenbezogene Daten erhoben und in pseudonymisierter (verschlüsselter) Form aufgezeichnet und gespeichert werden. Die Verarbeitung und Nutzung meiner Daten für die oben genannte Studie erfolgt ausschließlich wie in der Information auf secuTrail ®, auf der Webseite der Klinik für Neurologie des Klinikums rechts der Isar und im mir ausgehändigten Flyer beschrieben. Ich erteile meine Einwilligung in die Verarbeitung meiner personenbezogenen Daten, insbesondere auch meiner Gesundheitsdaten.

Meine Einwilligung ist freiwillig und kann jederzeit ohne Angabe von Gründen online auf der Plattform secuTrial® unter „Wiederruf meiner Einwilligung“ und ohne Nachteile widerrufen werden. Die Rechtmäßigkeit, der auf Grund der Einwilligung bis zum Widerruf erfolgten Verarbeitung wird hiervon nicht berührt. Beim Widerruf werden die auf Grundlage dieser Einwilligung gespeicherten Daten gelöscht oder anonymisiert, sofern dies gesetzlich zulässig ist.

Ich wurde über die Nutzung meiner Daten informiert und erteile im vorgenannten Rahmen meine Einwilligung. Ich hatte ausreichend Bedenkzeit und alle meine Fragen wurden zufriedenstellend beantwortet.

◯ Ich erkläre mich mit der Teilnahme an der Studie ParCoPa einverstanden. (1 - 210311)

◯ Ich erkläre mich mit der Teilnahme an der Studie ParCoPa NICHT einverstanden. (0 - 210312)

prelimbe {Horizontal-Radiobutton}

ParCoPa Fragebogen - Behandelnde mnpparcpbehand

{ IASHorizontalGroup: .... Standard - horizontal }

Vorbedingungen ist, daß der Befragte seine Einwilligung im vorherigen Formular bestätigt hat.

Vorbedingungen ist, daß der Befragte seine Einwilligung im vorherigen Formular "Volljährigkeit und Einwilligung" bestätigt hat.

{ **IASHeadline: .... Überschrift** }

{ **IASHorizontalGroup: .... Standard - horizontal** }

Hinweise zur Bedienung

Hinweise zur Bedienung

Bitte bedienen Sie die Anwendung unbedingt immer mit Klick (oder Tippen) auf eines der Bedienelemente (Menüeintrag, Button oder Antwortauswahl) innerhalb des Anwendungsfensters. Nutzen Sie NICHT die Bedienelemente des Browsers, nutzen Sie NICHT die Pfeiltasten oder wischen Sie NICHT, um zurück oder weiter zu navigieren.

Wenn möglich, starten Sie die Befragung mit dem Aufruf der Login-Seite immer über die Ihnen bekannte URL - auch wenn Sie sich über Abmelden bereits auf der Login-Seite befinden. Ein dafür eingerichtetes Lesezeichen im Browser ist dafür eine einfache Möglichkeit.

Den kleinen roten Stern können Sie ignorieren - er kennzeichnet lediglich Pflichteingabefelder. Ebenso können Sie das orange Warndreieck zwischen ZURÜCK und WEITER ignorieren - es zeigt lediglich an, dass es im gesamten Eingabeformular noch nicht beantwortete Fragen gibt.

Wenn Sie länger als 120 Minuten ohne eine Speichern-Aktion (entweder via FERTIG oder explizit über den EINGABE SPEICHERN-Button) in der Anwendung sind, wird Ihre Sitzung deaktiviert, d.h. Sie werden automatisch ausgeloggt. In diesem Fall sind all Ihre bis dahin nicht gespeicherten Eingaben verloren.

Sollte einmal (unerwarteterweise) die Ansicht so verändet (z.B. vergrößert) sein, dass der WEITER-Button nicht zu sehen ist, hilft oft ein Doppelklick auf die Anwendung, um die Ansichtsvergrößerung zurückzusetzen.

Oft macht es die Eingabe bequemer, wenn Sie das Browserfenster weniger hoch machen. Damit ist der WEITER-Button nicht so weit von den Antwort-Optionen entfernt.

{ **IASHorizontalGroup: .... Standard - horizontal** }

Hinweise zum Fragebogen

Hinweise zum Fragebogen

Im Fragebogen meinen wir mit SARS-CoV-2 das aktuell weltweit verbreitete Coronavirus und mit COVID-19 die durch dieses Virus hervorgerufene Erkrankung. Wenn wir Fragen zum Zeitraum VOR der Pandemie stellen, so meinen wir die Zeit VOR März 2020.

Ihr Fragebogen enthält 26 Fragen. Das Ausfüllen wird etwa 10 Minuten in Anspruch nehmen. Bitte achten Sie beim Ausfüllen von Freitextfeldern darauf, dass Sie keine Angaben machen, die einen Rückschluss auf Sie oder Dritte ermöglichen, also Sie beispielsweise keine Namen etc. angeben.

{ **IASHorizontalGroup: .... Standard - horizontal** }

1. Bitte geben Sie an, welche Spezialisierung Sie haben:

Kommentar

1. Bitte geben Sie an, welche Spezialisierung Sie haben.

- ◯ Neurologin/Neurologe oder Nervenarzt/ärztin (1 - 210196)
- ◯ Internist/Internistin (2 - 210197)
- ◯ Facharzt/ärztin für andere Fachrichtung (3 - 210198)

fb1 {Vertical-Radiobutton} **"1. Bitte geben Sie an, welche Spezialisierung Sie haben:"**

{ **IASHorizontalGroup: .... Standard - horizontal** }

2. Ich bin Hausarzt/Hausärztin

2. Ich bin Hausarzt/Hausärztin.

Kommentar

◯ Ja (1 - 210099) ◯ Nein (0 - 210118)

fb2 {Horizontal-Radiobutton} **"2. Ich bin Hausarzt/Hausärztin"**

{ **IASAlternateStandardGroup: .. Standard - vertikal, alternative Anordnung** }

3. In welchem Sektor der Patientenversorgung arbeiten Sie?

3. In welchem Sektor der Patientenversorgung arbeiten Sie?

Kommentar

◯ Praxis (1 - 210199)
◯ Rehabilitationsklinik (2 - 210200)
◯ Akutkrankenhaus (3 - 210201)
◯ Sonstiges: dann bitte ausfüllen (4 - 210202)

fb3 {Vertical-Radiobutton} **"3. In welchem Sektor der Patientenversorgung arbeiten Sie?"**

Bitte geben Sie den sonstigen Sektor ein.

fb3_x {Textfeld 40}

{ **IASHorizontalGroup: .... Standard - horizontal** }

4. Was trifft auf Sie am besten zu:

4. Was trifft auf Sie am besten zu?

Kommentar

◯ Ich behandele KEINE Parkinsonpatienten. (1 - 210203)
◯ Ich behandele SELTEN Parkinsonpatienten. (2 - 210204)
◯ Ich behandele REGELMAESSIG Parkinsonpatienten. (3 - 210205)
◯ Parkinsonpatienten sind mein SCHWERPUNKT, aber ich behandele auch andere Patienten. (4 - 210206)
◯ Ich behandele AUSSCHLIESSLICH Parkinsonpatienten. (5 - 210207)

fb4 {Vertical-Radiobutton} **"4. Was trifft auf Sie am besten zu:"**

{ **IASHorizontalGroup: .... Standard - horizontal** }

5. Wenn Sie Ihre Tätigkeit jetzt mit der Zeit VOR der Pandemie vergleichen: Wie oft haben Sie Parkinsonpatienten behandelt?

5. Wenn Sie Ihre Tätigkeit jetzt mit der Zeit VOR der Pandemie vergleichen: Wie oft haben Sie Parkinsonpatienten behandelt?

Kommentar

◯ Seltener (1 - 210208)
◯ Gleich oft (2 - 210209)
◯ Häufiger (3 - 210210)

fb5 {Vertical-Radiobutton} **"5. Wie oft haben Sie Parkinsonpatienten behandelt:"**

{ **IASHorizontalGroup: .... Standard - horizontal** }

6. Wenn Sie Ihre Tätigkeit jetzt mit der Zeit VOR der Pandemie vergleichen: Mussten Sie Termine Ihrer Patienten/innen auf Grund der Pandemie absagen?

6. Wenn Sie Ihre Tätigkeit jetzt mit der Zeit VOR der Pandemie vergleichen: Mussten Sie Termine Ihrer Patienten/innen auf Grund der Pandemie absagen?

Kommentar

◯ Nein (0 - 210118)
◯ Ja (wenige) (1 - 210211)
◯ Ja (viele) (2 - 210212)

◯ Ja (alle) (3 - 210213)
fb6 {Vertical-Radiobutton} "6. Mussten Sie Termine Ihrer Patienten/innen auf Grund der Pandemie absagen?"

{ IASHorizontalGroup: .... Standard - horizontal }
7. Wenn Sie Ihre Tätigkeit jetzt mit der Zeit VOR der Pandemie vergleichen: Haben Patienten/innen von sich aus auf Grund der Pandemie Termine abgesagt?

7. Wenn Sie Ihre Tätigkeit jetzt mit der Zeit VOR der Pandemie vergleichen: Haben Patienten/innen von sich aus auf Grund der Pandemie Termine abgesagt?

Kommentar

◯ Nein (0 - 210118)
◯ Ja (wenige) (1 - 210211)
◯ Ja (viele) (2 - 210212)
◯ Ja (alle) (3 - 210213)
fb7 {Vertical-Radiobutton} "7. Haben Patienten/innen von sich aus auf Grund der Pandemie Termine abgesagt?"

{ IASHorizontalGroup: .... Standard - horizontal }
8. Haben Sie das Gefühl Ihre Patienten/innen sind seit/während der Pandemie schlechter versorgt?

8. Wenn Sie Ihre Tätigkeit jetzt mit der Zeit VOR der Pandemie vergleichen: Haben Sie das Gefühl Ihre Patienten/innen sind während der Pandemie schlechter versorgt?

Kommentar

◯ Nein (0 - 210118)
◯ Ja (wenige) (1 - 210211)
◯ Ja (viele) (2 - 210212)
◯ Ja (alle) (3 - 210213)
fb8 {Vertical-Radiobutton} "8. Haben Sie das Gefühl Ihre Patienten/innen sind seit/während der Pandemie schlechter versorgt?"

{ IASHorizontalGroup: .... Standard - horizontal }
9. Wenn sie Ihre Tätigkeit jetzt mit der Zeit VOR der Pandemie vergleichen: Haben Sie das Gefühl Ihre Patienten/innen sind seit/während der Pandemie schlechter versorgt?

9. Wenn Sie Ihre Tätigkeit jetzt mit der Zeit VOR der Pandemie vergleichen: Haben Sie das Gefühl die Symptomatik Ihrer Patienten/innen verschlechtere sich wegen der Pandemie?

Kommentar

◯ Nein (0 - 210118)
◯ Ja (bei wenigen) (1 - 210308)
◯ Ja (bei vielen) (2 - 210309)
◯ Ja (bei allen) (3 - 210310)
fb9 {Vertical-Radiobutton} "9. Haben Sie das Gefühl die Symptomatik Ihrer Parkinson Patienten/innen verschlechtere sich wegen der Pandemie?"

{ IASAlternateStandardGroup: .. Standard - vertikal, alternative Anordnung }
10.1 Wenn Sie sich ein Bild über alle Ihre Parkinsonpatienten machen: Haben Sie den Eindruck, dass die Pandemie und die damit verbundenen Veränderungen zu einer Veränderung bestimmter Symptome geführt hat? Bitte wählen Sie jeweils die betreffende Antwortmöglichkeiten aus.

10. Wenn Sie sich ein Bild über alle Ihre Parkinsonpatienten machen: Haben Sie den Eindruck, dass die Pandemie und die damit verbundenen Veränderungen zu einer Veränderung bestimmter Symptome führte?

Kommentar

10.1 Motorische Symptome (Rigor, Tremor, Bradykinese, off-Zeit, Dyskinesien)

◯ Bei vielen verschlechtert (1 - 210214)
◯ Bei einigen verschlechtert (2 - 210215)
◯ Nicht verändert (3 - 210216)
◯ Bei einigen gebessert (4 - 210217)

◯ Bei vielen gebessert (5 - 210218)
fb10_1 {Vertical-Radiobutton} **"Motorische Symptome (Rigor, Tremor, Bradykinese, off-Zeit, Dyskinesien)"**

10.2 Neuropsychiatrische Symptome (Depressivität, Angst, Zwangsverhalten, Halluzinationen)

◯ Bei vielen verschlechtert (1 - 210214)
◯ Bei einigen verschlechtert (2 - 210215)
◯ Nicht verändert (3 - 210216)
◯ Bei einigen gebessert (4 - 210217)
◯ Bei vielen gebessert (5 - 210218)
fb10_2 {Vertical-Radiobutton} **"Neuropsychiatrische Symptome (Depressivität, Angst, Zwangsverhalten, Halluzinationen)"**

10.3 Autonome Symptome (Obstipation, Urininkontinenz, orthostatische Dysregulation)

◯ Bei vielen verschlechtert (1 - 210214)
◯ Bei einigen verschlechtert (2 - 210215)
◯ Nicht verändert (3 - 210216)
◯ Bei einigen gebessert (4 - 210217)
◯ Bei vielen gebessert (5 - 210218)
fb10_3 {Vertical-Radiobutton} **"Autonome Symptome (Obstipation, Urininkontinenz, orthostatische Dysregulation)"**

10.4 Schlafstörungen

◯ Bei vielen verschlechtert (1 - 210214)
◯ Bei einigen verschlechtert (2 - 210215)
◯ Nicht verändert (3 - 210216)
◯ Bei einigen gebessert (4 - 210217)
◯ Bei vielen gebessert (5 - 210218)
fb10_4 {Vertical-Radiobutton} **"Schlafstörungen"**

{ **IASAlternateStandardGroup: .. Standard - vertikal, alternative Anordnung** }

11.1 Hatten Sie Probleme, während der Pandemie folgende Therapien für Ihre Patienten/innen zu organisieren? Bitte wählen Sie jeweils die betreffende Angabe aus.

Kommentar

11. Hatten Sie Probleme, während der Pandemie folgende Therapien für Ihre Patienten/innen zu organisieren?

11.1 Rehabilitative Maßnahmen

◯ Keine Probleme (1 - 210219)
◯ Selten Probleme (2 - 210220)
◯ Oft Probleme (3 - 210221)
◯ Unmöglich (4 - 210222)
fb11_1 {Vertical-Radiobutton} **"Rehabilitative Maßnahmen"**

11.2 Physio-/Ergotherapie

◯ Keine Probleme (1 - 210219)
◯ Selten Probleme (2 - 210220)
◯ Oft Probleme (3 - 210221)
◯ Unmöglich (4 - 210222)
fb11_2 {Vertical-Radiobutton} **"Physio-/Ergotherapie"**

11.3 Logopädie

◯ Keine Probleme (1 - 210219)
◯ Selten Probleme (2 - 210220)
◯ Oft Probleme (3 - 210221)
◯ Unmöglich (4 - 210222)
fb11_3 {Vertical-Radiobutton} **"Logopädie"**

11.4 Endoskopische Untersuchungen (z.B.: Implantation einer Jejunal-Sonde)

◯ Keine Probleme (1 - 210219)
◯ Selten Probleme (2 - 210220)
◯ Oft Probleme (3 - 210221)
◯ Unmöglich (4 - 210222)
fb11_4 {Vertical-Radiobutton} **"Endoskopische Untersuchungen (z.B.: Implantation einer Jejunal-Sonde)"**

11.5 DBS-Operation (Tiefe Hirnstimulation)

◯ Keine Probleme (1 - 210219)
◯ Selten Probleme (2 - 210220)
◯ Oft Probleme (3 - 210221)
◯ Unmöglich (4 - 210222)
fb11_5 {Vertical-Radiobutton} **"DBS-Operation (Tiefe Hirnstimulation)"**

11.6 Krankenhauseinweisung

◯ Keine Probleme (1 - 210219)
◯ Selten Probleme (2 - 210220)
◯ Oft Probleme (3 - 210221)
◯ Unmöglich (4 - 210222)
fb11_6 {Vertical-Radiobutton} **"Krankenhauseinweisung"**

{ **IASHorizontalGroup: .... Standard - horizontal** }
12. Mussten Sie Ihre Praxis/Abteilung (teilweise) durch die Pandemie schließen?

Kommentar

12. Mussten Sie Ihre Praxis/Abteilung (teilweise) durch die Pandemie schließen?

◯ Nein (0 - 210118) ◯ Ja (teilweise) (1 - 210223) ◯ Ja (komplett) (3 - 210224)
fb12 {Horizontal-Radiobutton} **"12. Mussten Sie Ihre Praxis/Abteilung (teilweise) durch die Pandemie schließen?"**

{ **IASHorizontalGroup: .... Standard - horizontal** }
13. Mussten Sie auf Grund der Pandemie finanzielle Einbußen hinnehmen?

Kommentar

13. Mussten Sie auf Grund der Pandemie finanzielle Einbußen hinnehmen?

◯ Keine (0 - 210225) ◯ Geringere (1 - 210226) ◯ Deutliche (2 - 210227)
fb13 {Horizontal-Radiobutton} **"13. Mussten Sie auf Grund der Pandemie finanzielle Einbußen hinnehmen?"**

{ **IASHorizontalGroup: .... Standard - horizontal** }
14. Mussten Sie auf Grund der Pandemie Investitionen tätigen?

Kommentar

14. Mussten Sie auf Grund der Pandemie Investitionen tätigen?

◯ Keine (0 - 210225) ◯ Geringere (1 - 210226) ◯ Deutliche (2 - 210227)
fb14 {Horizontal-Radiobutton} **"14. Mussten Sie auf Grund der Pandemie Investitionen tätigen?"**

{ IASHorizontalGroup: .... Standard - horizontal }

15. Bedeuten Hygienekonzepte in Ihrer Abteilung/Praxis Mehrarbeit für Sie?

15. Bedeuten Hygienekonzepte in Ihrer Abteilung/Praxis Mehrarbeit für Sie?

Kommentar

◯ Keine (0 - 210225) ◯ Geringere (1 - 210226) ◯ Deutliche (2 - 210227)

fb15 {Horizontal-Radiobutton} **"15. Bedeuten Hygienekonzepte in Ihrer Abteilung/Praxis Mehrarbeit für Sie?"**

{ IASAlternateStandardGroup: .. Standard - vertikal, alternative Anordnung }

16.1 Haben Sie Ihr Angebot durch die Pandemie erweitert? Beispielsweise durch:

16.1 Haben Sie Ihr Angebot aufgrund der Pandemie erweitert?

Kommentar

Durch vermehrte häusliche Konsultationen

◯ Ja (1 - 210099) ◯ Nein (0 - 210118)

fb16_1 {Horizontal-Radiobutton}

17.1 Wie wurden diese Angebote von Ihren Parkinsonpatienten genutzt?

◯ Nicht genutzt (1 - 210232)
◯ Wenig genutzt (2 - 210233)
◯ Viel genutzt (3 - 210234)

fb17_1 {Vertical-Radiobutton} **"Vermehrte häusliche Konsultationen"**

{ IASAlternateStandardGroup: .. Standard - vertikal, alternative Anordnung }

16.2 Haben Sie Ihr Angebot durch die Pandemie erweitert? Beispielsweise durch:

16.2 Haben Sie Ihr Angebot aufgrund der Pandemie erweitert?

Kommentar

Durch Online-Angebote/Sprechstunden

◯ Ja (1 - 210099) ◯ Nein (0 - 210118)

fb16_2 {Horizontal-Radiobutton}

17.2 Wie wurden diese Angebote von Ihren Parkinsonpatienten genutzt?

◯ Nicht genutzt (1 - 210232)
◯ Wenig genutzt (2 - 210233)
◯ Viel genutzt (3 - 210234)

fb17_2 {Vertical-Radiobutton} **"Online-Angebote/Sprechstunden"**

{ IASAlternateStandardGroup: .. Standard - vertikal, alternative Anordnung }

16.3 Haben Sie Ihr Angebot durch die Pandemie erweitert? Beispielsweise durch:

16.3 Haben Sie Ihr Angebot aufgrund der Pandemie erweitert?

Kommentar

Durch telefonische Sprechstunden

◯ Ja (1 - 210099) ◯ Nein (0 - 210118)

fb16_3 {Horizontal-Radiobutton}

17.3 Wie wurden diese Angebote von Ihren Parkinsonpatienten genutzt?

◯ Nicht genutzt (1 - 210232)
◯ Wenig genutzt (2 - 210233)
◯ Viel genutzt (3 - 210234)

fb17_3 {Vertical-Radiobutton} **"Telefonische Sprechstunden"**

{ IASAlternateStandardGroup: .. Standard - vertikal, alternative Anordnung }

16.4 Haben Sie Ihr Angebot durch die Pandemie erweitert? Beispielsweise durch:

Kommentar

16.4 Haben Sie Ihr Angebot aufgrund der Pandemie erweitert?

Durch schriftliche Informationen für Ihre Patienten/innen

◯ Ja (1 - 210099) ◯ Nein (0 - 210118)

fb16_4 {Horizontal-Radiobutton}

17.4 Wie wurden diese Angebote von Ihren Parkinsonpatienten genutzt?

◯ Nicht genutzt (1 - 210232)
◯ Wenig genutzt (2 - 210233)
◯ Viel genutzt (3 - 210234)

fb17_4 {Vertical-Radiobutton} **"Schriftliche Informationen für Ihre Patienten/innen"**

{ IASAlternateStandardGroup: .. Standard - vertikal, alternative Anordnung }

16.5 Haben Sie Ihr Angebot durch die Pandemie erweitert? Beispielsweise durch:

Kommentar

16.5 Haben Sie Ihr Angebot aufgrund der Pandemie erweitert?

Durch andere Maßnahmen

◯ Ja (1 - 210099) ◯ Nein (0 - 210118)

fb16_5 {Horizontal-Radiobutton}

Bitte benennen Sie die Maßnahmen

fb16_x {Textfeld 40}

17.5 Wie wurden diese Angebote von Ihren Parkinsonpatienten genutzt?

◯ Nicht genutzt (1 - 210232)
◯ Wenig genutzt (2 - 210233)
◯ Viel genutzt (3 - 210234)

fb17_5 {Vertical-Radiobutton} **"Andere Maßnahmen"**

{ IASHorizontalGroup: .... Standard - horizontal }

18. Haben Sie selbst an COVID-19 Erkrankte behandelt/visitiert?

Kommentar

18. Haben Sie selbst an COVID-19 Erkrankte behandelt/visitiert?

◯ Nein (0 - 210118) ◯ Ja (1 - 210099)

fb18 {Horizontal-Radiobutton} **"18. Haben Sie selbst an Covid-19 Erkrankte behandelt/visitiert?"**

{ IASHorizontalGroup: .... Standard - horizontal }

19. Fühlen Sie sich gesundheitlich gefährdet während Ihrer Arbeit?

Kommentar

19. Fühlen Sie sich gesundheitlich gefährdet während Ihrer Arbeit?

◯ Nein (0 - 210118) ◯ Ja (1 - 210099)

fb19 {Horizontal-Radiobutton} **"19. Fühlen Sie sich gesundheitlich gefährdet während Ihrer Arbeit?"**

{ IASHorizontalGroup: .... Standard - horizontal }

20. Fühlen Sie sich durch die Ihnen zur Verfügung stehenden Schutzmaßnahmen geschützt? Nein; ja (ein wenig geschützt); ja (sehr gut geschützt)

Kommentar

20. Fühlen Sie sich durch die Ihnen zur Verfügung stehenden Schutzmaßnahmen geschützt?

◯ Nein (0 - 210118) ◯ Ja (ein wenig geschützt) (1 - 210228) ◯ Ja (sehr gut geschützt) (2 - 210229)
fb20 {Horizontal-Radiobutton} **"20. Fühlen sie sich durch die Ihnen zur Verfügung stehenden Schutzmaßnahmen geschützt?"**

{ **IASHorizontalGroup: .... Standard - horizontal** }
21. Haben Sie Ihre Arbeit mit Parkinson Patienten/innen während der Pandemie als stressiger/fordernder im Vergleich zu der Zeit vor der Pandemie empfunden?

Kommentar

21. Haben Sie Ihre Arbeit mit Parkinsonpatienten während der Pandemie als stressiger/fordernder im Vergleich zu der Zeit vor der Pandemie empfunden?

◯ Nein (0 - 210118) ◯ Ja (1 - 210099)
fb21 {Horizontal-Radiobutton} **"21. Haben Sie Ihre Arbeit mit Parkinsonpatienten während der Pandemie als stressiger/fordernder im Vergleich zu der Zeit vor der Pandemie empfunden?"**

{ **IASHorizontalGroup: .... Standard - horizontal** }
22. Hat sich Ihre Behandlungsqualität subjektiv verschlechtert?

Kommentar

22. Hat sich Ihre Behandlungsqualität subjektiv verschlechtert?

◯ Nein (0 - 210118) ◯ Ja (ein wenig) (1 - 210230) ◯ Ja (deutlich) (2 - 210231)
fb22 {Horizontal-Radiobutton} **"22. Hat sich Ihre Behandlungsqualität subjektiv verschlechtert?"**

{ **IASHorizontalGroup: .... Standard - horizontal** }
23. Sind Sie gegen Influenza geimpft?

Kommentar

23. Sind Sie gegen Influenza geimpft?

◯ Ja (1 - 210099) ◯ Nein (0 - 210118)
fb23 {Horizontal-Radiobutton} **"23. Sind Sie gegen Influenza geimpft?"**

{ **IASHorizontalGroup: .... Standard - horizontal** }
24. Würden Sie sich, sollte ein Impfstoff gegen SARS-CoV-2 verfügbar sein, impfen lassen?

Kommentar

24. Würden Sie sich, sollte ein Impfstoff gegen SARS-CoV-2 verfügbar sein, impfen lassen?

◯ Ja (1 - 210099) ◯ Nein (0 - 210118)
fb24 {Horizontal-Radiobutton} **"24. Würden Sie sich, sollte ein Impfstoff gegen COVID-19 verfügbar sein, impfen lassen?"**

{ **IASAlternateStandardGroup: .. Standard - vertikal, alternative Anordnung** }
25. Warum würden Sie sich nicht impfen lassen?

Kommentar

25. Warum würden Sie sich nicht impfen lassen?

◯ Ich finde impfen generell nicht sinnvoll (1 - 210302)
◯ Ich denke nicht, dass ich mich an COVID-19 anstecken könnte (2 - 210303)
◯ Ich vertraue einem neuen Impfstoff noch nicht (3 - 210304)
◯ Ich habe Angst vor Nebenwirkungen (4 - 210305)
◯ Andere Gründe (5 - 210306)
fb25 {Vertical-Radiobutton} **"25. Warum würden Sie sich nicht impfen lassen?"**

fb25_x {Textfeld 40} **"Andere Gründe sich nicht impfen zu lassen"**

{ **IASHorizontalGroup: .... Standard - horizontal** }
26. Würden Sie Ihren Parkinson-Patienten empfehlen, sich impfen zu lassen?

Kommentar

26. Würden Sie Ihren Parkinson-Patienten empfehlen, sich impfen zu lassen?

◯ Ja (1 - 210099) ◯ Nein (0 - 210118)
fb26 {Horizontal-Radiobutton} **"26. Würden Sie Ihren Parkinson-Patienten empfehlen, sich impfen zu lassen?"**

{ **IASHorizontalGroup: .... Standard - horizontal** }
Kontakt bei Folgebefragungen
Kontakt bei Folgebefragungen

Wir bedanken uns ganz herzlich für Ihre Mitarbeit bei der ParCoPa Studie! Mit Ihren Angaben helfen Sie uns, besser zu verstehen, welche Auswirkungen die COVID-19-Pandemie auf Menschen mit M. Parkinson, ihre Angehörigen und Behandler hat.

Wenn Sie über Folgebefragungen informiert werden wollen, dann **füllen Sie bitte noch das folgende Formular „NEUERLICHE KONTAKTAUFNAHME“ aus**.

Neuerliche Kontaktaufnahme - Behandelnde mnpparcpkontaktb

{ **IASAlternateStandardGroup: .. Standard - vertikal, alternative Anordnung** }
Ich willige ein, dass ich von externen Datentreuhänder erneut kontaktiert werden darf, um über die Starts der bis zu zwei Folgebefragungen im Rahmen dieses Forschungsprojekts zu erhalten.
Ich willige ein, dass ich von einem unabhängigen Datentreuhänder erneut kontaktiert werden darf, um an bis zu zwei Folgebefragungen im Rahmen dieses Forschungsprojektes erinnert zu werden.

Kommentar

◯ Ja (1 - 210099) ◯ Nein (0 - 210118)
kontjn {Horizontal-Radiobutton} **"Neuerliche Kontaktaufnahme: Ich willige ein, dass ich von einem unabhängigen Datentreuhänder erneut kontaktiert werden darf, um an bis zu zwei Folgebefragungen im Rahmen dieses Forschungsprojektes erinnert zu werden."**

Bitte geben Sie hier Ihre E-Mail-Adresse ein. (max. 60 Zeichen)

kontmail {Textfeld 60}

{ **IASHorizontalGroup: .... Standard - horizontal** }
Die Befragung ist abgeschlossen, Sie können sich nach FERTIG abmelden.
Vielen Dank für Ihre Teilnahme an der ParCoPa-Befragung!

Ihre Angaben werden uns helfen, die Situation von Patienten und Patientinnen mit M. Parkinson während der Pandemie besser zu verstehen.

Nachdem Sie auf FERTIG gedrückt haben, können Sie sich im Hauptmenü —> **Abmelden** .

Widerruf der Einwilligung

Widerruf der Einwilligung mnpparcpwiderruf

{ **IASHorizontalGroup: .... Standard - horizontal** }
Hiermit ziehe ich meine Einwilligung zurück.
Hiermit ziehe ich meine Einwilligung zurück.

Hilfe
Kommentar

Wenn Sie mit Ja antworten, wird die Studienleitung benachrichtigt und veranlassen, dass all Ihre bereits eingegebenen Befragungsdaten gelöscht

werden. Danach wird Ihre Befragtenkennung (Pseudonym) ebenfalls gelöscht, sodass Sie sich nicht mehr damit anmelden können. Der unabhängige Datentreuhänder wird Ihre Emailadresse, soweit Sie eine angegeben haben, ebenfalls löschen. Eine Teilnahme an Folgeumfragen ist dann nicht mehr möglich.

◯ Ja (1 - 210099) ◯ Nein (0 - 210118)
widerrjn {Horizontal-Radiobutton} **"Hiermit ziehe ich meine Einwilligung zurück."**
